# Case Report: Extracorporeal Membrane Oxygenation Followed by Intra-Aortic Balloon Counterpulsation Successfully Treated Cardiac Arrest Caused by Anomalous Origin of a Left Coronary Artery From the Right Coronary Sinus

**DOI:** 10.3389/fmed.2022.936721

**Published:** 2022-06-29

**Authors:** Xiaolan Xu, Peng Xu, Xiaoyan Wu, Hua Lin, Yinhua Chen, Xiaohua Hu, Jiangquan Yu, Ruiqiang Zheng

**Affiliations:** ^1^Department of Intensive Care Medicine, Northern Jiangsu People’s Hospital, Clinical Medical College of Yangzhou University, Yangzhou, China; ^2^Department of Hepatobiliary and Pancreatic Surgery, Northern Jiangsu People’s Hospital, Clinical Medical College of Yangzhou University, Yangzhou, China; ^3^Department of Echocardiography, Northern Jiangsu People’s Hospital, Clinical Medical College of Yangzhou University, Yangzhou, China; ^4^Department of Radiology, Northern Jiangsu People’s Hospital, Clinical Medical College of Yangzhou University, Yangzhou, China

**Keywords:** ECMO, IABP, cardiac arrest, AOCA, case report

## Abstract

**Background:**

Anomalous origin of a coronary artery (AOCA) is defined as the failure of the coronary artery to originate from the normal coronary sinus. The anomalous origin of the left coronary artery arising from the right coronary sinus is rare, dangerous and at risk of malignant arrhythmia, sudden death, and high mortality.

**Case Presentation:**

In this study, we present a 14-year-old adolescent male who went to a hospital with transient unconsciousness after exercise, who subsequently developed cardio arrest due to malignant arrhythmia. He was admitted to the intensive care unit, and who subsequently received successful veno-arterial extracorporeal membrane oxygenation (VA ECMO) assisted circulation followed by intra-aortic balloon counterpulsation (IABP). Echocardiography and cardiac CTA were also performed, further confirming that the abnormal left coronary artery originated from the right coronary sinus. The patient subsequently underwent heart surgery.

**Conclusion:**

The successful treatment of the patient in this report was attributed to the immediately VA ECMO, supplemented by IABP. Establishing clear diagnosis is a process of multidisciplinary joint diagnosis, which provides a reference for clinicians when encountering similar cases.

## Introduction

Anomalous origin of a coronary artery (AOCA) is generally caused by abnormal or incomplete development of coronary artery during the embryonic period. The overall detection rate of AOCA is approximately 0.6–1.3% (1, 2). Abnormal origin of left coronary artery from the right sinus is significantly rare (3), but it is frequently associated with early cardiac death, especially during vigorous exercise.

In this study, the patient was admitted to a hospital with transient unconsciousness during exercise, subsequently developed cardio arrest due to malignant arrhythmia, and was admitted to the intensive care unit (ICU). Veno-arterial extracorporeal membrane oxygenation (VA ECMO) followed by intra-aortic balloon counterpulsation (IABP) successfully saved the patient’s life. He was finally diagnosed with AOCA, and later underwent follow-up treatment. ECMO, sequential IABP successfully rescued the cardiac arrest and cardiogenic shock caused by abnormal coronary origin (the left coronary artery originates from the right coronary sinus with an initial interarterial course) was rarely reported.

## Case Presentation

**FIGURE 1:**
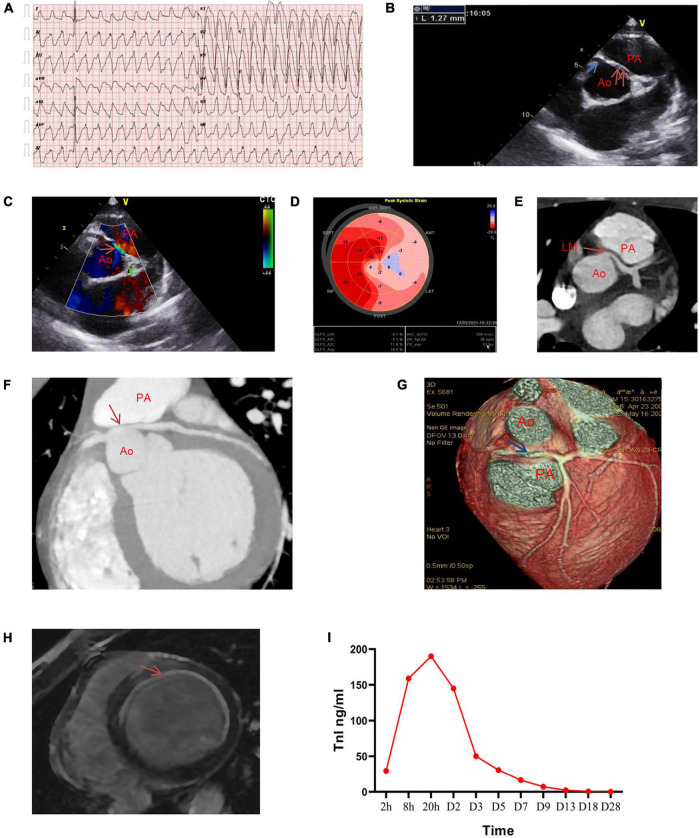
Clinical images before surgery. **(A)** Emergency electrocardiogram revealing wide QRS complex tachycardia. **(B–D)** Images of transthoracic echocardiography on HD20 showing staged wall motion hypokinesia (extensive anterior wall, left ventricular lateral wall and apex), LVEF, 45%. **(B)** Parasternal short-axis view of echocardiography shows that the right coronary artery (blue single arrow) opens in the right coronary sinus, with an inner diameter of approximately 3.3 mm, and the left coronary artery (red double arrow) opens in the right coronary sinus and between the aorta and the pulmonary artery. The inner diameter of the opening is approximately 1.27 mm. **(C)** Acceleration of blood flow can be observed in the interarterial left main (LM) coronary artery (red arrow). **(D)** Ultrasound speckle tracking image shows the longitudinal strain of the anterior septum, left ventricular anterior wall, apex, anterior lateral wall, and posterior wall (left coronary artery area) decreases during systoling, whereas the posterior septum and left ventricular inferior wall (right coronary artery area) is normal. Left ventricular global longitudinal strain (GLS), –10%. **(E–G)** Images of coronary computed tomography angiography (CTA). Cross-sectional view of cardiac **(E)**, Maximum intensity projection (MIP) image **(F)**, and Volume-rendered (VR) image of the cardiac **(G)** revealing the left coronary artery originating from the right coronary sinus and coursing in an inter-arterial manner, with evident stenosis after being squeezed by the aorta and pulmonary artery. **(H)** Contrast-enhanced cardiovascular magnetic resonance image (MRI) reveals abnormal enhancement of left ventricular septum, anterior wall, lateral wall, considering the changes in subendocardial myocardial infarction. **(I)** Dynamic evolution of troponin I during hospitalization in the patient. LVEF, left ventricular ejection fraction; Ao, aorta; PA, pulmonary artery.

A 14-year-old adolescent male was admitted to the intensive care unit (ICU) of our hospital due to sudden “transient unconsciousness while running 2 h ago.” At that time, the patient was pale and sweating profusely, which resolved spontaneously after 2 min. After waking, he complained of chest tightness and cold limbs, and went to the emergency room. At that time, his blood pressure was 95/60 mmHg, heart rate 160/min, with ventricular tachycardia, respiratory rate 34/min. The emergency doctor gave amiodarone for anti-arrhythmic. Approximately half an hour later, the patient suddenly experienced ventricular fibrillation, loss of consciousness with a Glasgow Coma Score of 4 (GCS: E1, M1, V2). Immediately chest compressions, defibrillation, graded intravenous push of epinephrine, and tracheal intubation were performed. After cardiopulmonary resuscitation (CPR), the patient’s blood pressure was 83/53 mmHg with a norepinephrine infusion of 2 ug/min.kg pump and lidocaine for antiarrhythmic. He had experienced syncope during exercise two times and both were relieved after rest without medical attention when he was 12 years old. There was no family history of sudden death. Emergency laboratory results were as follows: hemoglobin, 126 g/L, white blood cells, 11.55 × 10^9^/L, platelet, 208 × 10^9^/L, total bilirubin, 18.4 umol/L, alanine transaminase, 702 u/L, aspartate transaminase, 1197 u/L, creatinine, 143 umol/L, glucose, 17.5 mmol/L, lactate, 10.9 mmol/L, bicarbonate, 14.6 mmol/L, troponin I, 29.5 ng/ML, myoglobin, 18680 ng/ML, creatine kinase-MB, 501 u/L, N-terminal pro-BNP, 1430 pg/ML. An electrocardiogram showed a wide QRS complex tachycardia (Figure 1A). Emergency point-of-care ultrasound prompted: extremely weak heart contraction. Considering that the patient would suffer cardiac arrest again at any time, he was immediately admitted to the ICU.

When the ventilator with PEEP 16 cmH20, the patient still had a large amount of pink foamy sputum gushing out of the tracheal tube, and extensive blister sounds could be heard by stethoscope in both lungs. Simultaneously, he was anuria. High-dose vasopressor drugs (maximum norepinephrine, 3 ug/kg.min combinated epinephrine, 0.1 ug/kg.min [intravenously pumped]) were administered to maintain circulation. Fast echocardiography revealed diffusely weakened full ventricular wall motion and left ventricular ejection fraction (LVEF) of 20%. The patient was a student in the third year of junior high school, preparing for the high school entrance examination and was in a state of fatigue. He had no high-risk factors for myocardial infarction such as diabetes and coronary heart disease. Although he had no signs and symptoms of recent viral upper respiratory infection or enteroviral infection, he was diagnosed with acute fulminant myocarditis (FM), cardiogenic shock, malignant arrhythmias, cardiac arrest and post-CPR, multiple organ dysfunction syndrome, acute pulmonary edema, acute renal injury, acute hepatic dysfunction, and metabolism acidosis.

Considering his rapid clinical deterioration, bedside veno-arterial extracorporeal membrane oxygenation (VA ECMO) was implanted (right internal jugular vein-right femoral artery), and initial ECMO centrifugal pump speed was 3300 rpm which supported flow rate 3.0 L/min. The heat exchanger was setted 35.5°C to keep the patient’s blood temperature was less than 36°C for brain resuscitation. The distal perfusion catheter was placed which directed a proportion of the returned oxygenated blood flow from the ECMO circuit to the distal of the right femoral artery to prevent limb ischemia. Considering the patient was volume overload, anuria, severe metabolic acidosis with acute renal injury, continuous renal replacement therapy (CRRT) was connected to the ECMO to provided solute depuration, fluid removal, and control of electrolyte and acid-base balance. The bleeding end of CRRT was connected behind the membrane of ECMO, and returned to the front of the ECMO membrane. Continuous venovenous hemofiltration (CVVH) with predominantly convective solute clearance was used. The formulation of CRRT was adjusted according to the blood gas results. He was administered high-dose methylprednisolone, concomitant antiviral therapy with acyclovir, and systemic anticoagulation with argatroban.

With the help of ECMO, the doses of vasopressor drugs were gradually reduced. After 20 h of ECMO, the patient became conscious and urinated with a urine output of 50 ml/h. We used remifentanil for pain relief and propofol for sedation, and we deactivated CRRT on hospital day (HD)3. By HD5, the patient’s cardiac systolic function was progressively enhanced, and daily bedside echocardiography showed that LVEF gradually increased to 47% under the condition of ECMO flow rate of 2 L/min with 0.1 ug/min.kg of norepinephrine intravenously pumped. Therefore, we reduced the flow rate of ECMO to 1 L/min. There was no significant change in the patient’s vasopressors for about 1 h, and ECMO was weaned. There was no significant change in the vasopressor drugs and LVEF after weaned from ECMO. Ventilator condition was pressure support ventilation (PSV) with the PEEP 6 cmH2O on HD6, so we removed the tracheal intubation. On the night of HD6, the patient complained of chest tightness and experienced cardiogenic shock again, while his blood pressure decreased, as low as 55/32 mmHg (0.3 ug/min.kg of norepinephrine combinated epinephrine 0.1 ug/min.kg). Bedside echocardiography revealed left ventricular filling, and his cardiac systolic function (LVEF, 38%) was worse than before (LVEF, 46% by HD6 before extubation). Thus, we placed IABP to support circulation. After the use of IABP, the patient’s circulation improved again, the dose of vasopressor drugs was reduced. Repeat bedside echocardiography revealed that his cardiac function (LVEF, 46%) had improved. Therefore, we gradually reduced the IABP counterpulsation ratio from 1:1 to 1:3. At HD12, the IABP counterpulsation was stopped for 1 h. The patient had no chest tightness, normal urine output, no drop in blood pressure (0.05 ug/min.kg of norepinephrine), blood gas suggested no metabolic acidosis and IABP was removed.

The patient’s vital signs were stable and there was no vasopressors on HD20. But his activity tolerance remained poor, and bedside echocardiography: left ventricular systolic function was still weak, and other cardiac functions were normal. Therefore, we decided to take the patient out of the ICU to further search for his underlying cause. Considering that he was a juvenile patient and suffered cardiac arrest after exercising, we began to pay attention to the coronary origin. Echocardiography on HD20 (Figures 1B–D) revealed LVEF, 45%. The left main (LM) coronary artery originated from the right coronary sinus with an initial interarterial course between the aorta and pulmonary artery, which was obviously compressed by the aorta especially during systole. The opening of the LM was narrowed with a diameter of 1.27 mm, and the blood flow of interarterial course was accelerated. Myocardial systolic longitudinal strain in the area of the left coronary artery was decreased (left ventricular global longitudinal strain [GLS], −10%). Coronary computed tomography angiography (CTA) (Figures 1E–G) revealed the varied origin of the left coronary artery, running between the aorta and pulmonary artery, and evident stenosis after being squeezed by the aorta and pulmonary artery. Contrast-enhanced cardiovascular magnetic resonance image (MRI) (Figure 1H) revealed abnormal enhancement of the left ventricular endocardium, suggesting subendocardial myocardial infarction. The patient’s troponin I was up to 190 ng/ml (at 20 hours of onset), and then gradually decreased to normal level. Figure 1I showed dynamic evolution of troponin I during the patient’s hospitalization.

The patient was diagnosed with congenital left coronary artery anomalies originating from the right coronary sinus with an interarterial course between the aorta and pulmonary artery, acute left ventricular subendocardial infarction, cardiogenic shock, malignant arrhythmias, cardiac arrest and post-CPR, multiple organ dysfunction syndrome, acute pulmonary edema, acute renal failure, acute hepatic dysfunction, and metabolism acidosis.

**FIGURE 2:**
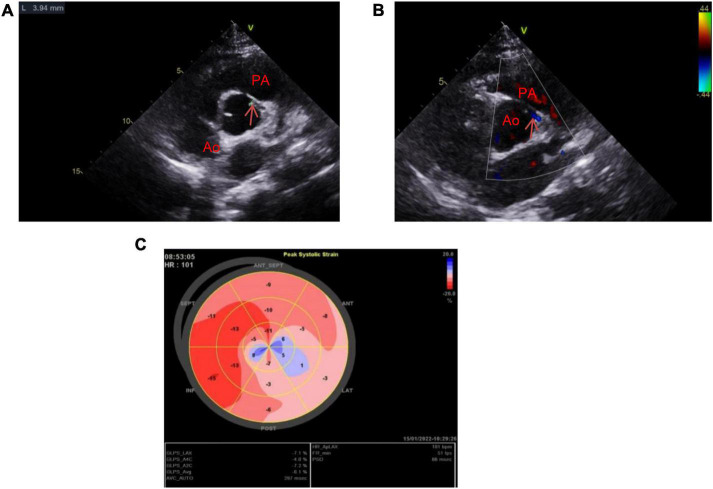
Images of transthoracic echocardiography 1 month post-operatively illustrating staged ventricular wall motion hypokinesia (extensive anterior wall, left ventricular lateral wall, and apex) and LVEF, 40%. **(A)** After unroofing of the intramural portion, the diameter of the left coronary ostium is approximately 3.94 mm. **(B)** The LM blood flow is in normal velocity. **(C)** Ultrasound speckle tracking image shows GLS of the left ventricle, –6.1%.

**FIGURE 3:**
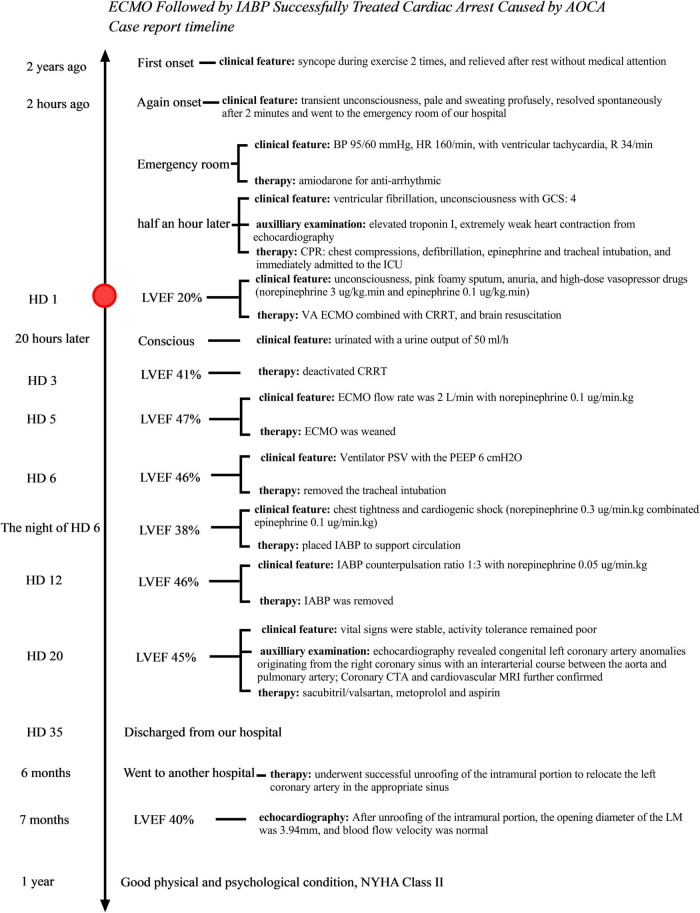
The timeline of the case.

The patient had cardiac insufficiency, and New York Heart Association (NYHA) Class II. Therefore, long-term, restorative strategies for neuroendocrine inhibition with sacubitril/valsartan, metoprolol, and aspirin for platelet aggregation were administered therapeutically. On HD35, the patient was discharged, and follow-up treatment focused on improving myocardial remodeling and restoring cardiac function. Six months later, he underwent successful unroofing of the intramural portion in another hospital to relocate the left coronary artery in the appropriate sinus. Echocardiography 1 month post-operatively (Figures 2A–C) revealed LVEF, 40%, the opening diameter of the LM was 3.94 mm, and blood flow velocity was normal. Moreover, the GLS of the left ventricle was 6.1%. At follow-up, the patient came to our hospital for reexamination about once a month. He was in good physical and psychological condition. He had been taking oral sacubitril/valsartan, metoprolol, and aspirin. He was asymptomatic at rest, and his physical activity was mildly restricted (NYHA Class II cardiac function). The patient is currently suspended from school for recuperation, and at the same time studies moderately for 2 h a day, and is ready to return to school in the second half of the year. The progress and decision-making of the case above are reflected in the timeline (Figure 3).

## Discussion

Veno-arterial extracorporeal membrane oxygenation is a rescue therapy that can stabilize patients with hemodynamic compromise, with or without respiratory failure, for days or weeks (4). It is not uncommon to use ECMO, IABP, or a combination of both to rescue patients from cardiac arrest. But most of the studies were based on patients with cardiac arrest caused by acute coronary syndrome or acute fulminant myocarditis (5). We reported a case of ECMO followed by IABP successfully treated cardiac arrest and cardiogenic shock caused by AOCA.

The 14-year-old patient had cardiac arrest in the emergency room, and was in extremely critical state. In cardiology, the main indications for ECMO include cardiac arrest, cardiogenic shock, post-cardiotomy shock, refractory ventricular tachycardia, and acute management of complications of invasive procedures (4). Therefore, he had an absolute indication of ECMO. Considering the patient was volume overload, anuria, severe metabolic acidosis with acute renal injury, CRRT was connected to the ECMO. Among patients with cardiac arrest and ECMO support, those receiving CRRT tended to be more critically ill and had reduced survival (6). However, this patient had a good survival outcome, and it can provide the clinician with the treatment experience of ECMO combined with CRRT. On the night of the extubation, the patient experienced cardiogenic shock again. The reason could be without PEEP after extubation, the patient’s blood volume returned to the heart was increased more than before. IABP can reduce afterload, increase cardiac output, optimize coronary flow and decrease oxygen consumption. It has been used to improve hemodynamic parameters in patients with cardiogenic shock for more than four decades (7). So the placement of IABP supported the patient’s circulation.

The most common cause of cardiac arrest and cardiogenic shock was acute myocardial infarction, and a small part of the cause was acute FM, especially in young people (8). Fulminant myocarditis (FM) is an uncommon syndrome characterized by sudden and severe diffuse cardiac inflammation often leading to death resulting from cardiogenic shock, ventricular arrhythmias, or multiorgan system failure (9). In the real-world practice the diagnosis of FM has undoubtedly shifted from being mainly biopsy-based to cardiac magnetic resonance imaging-based (CMRI-based) for characterizing myocardial tissue changes and the measurement of high-sensitive troponin levels for identifying myocardial injury in most of clinical scenarios (10). But CMRI is not the initial diagnostic technique in patients in critical conditions (10). This patient was a 14-year-old teenager with no previous high-risk factors for myocardial infarction such as diabetes and coronary heart disease. In the pre-morbidity, he was tired due to preparing for the senior high school entrance examination. He was running at the time of onset. After resuscitation, arrhythmia was manifested as unsustainable circulation, severe cardiogenic shock, and elevated troponin level. The electrocardiogram showed wide QRS tachycardia, and there was no clear coronary location. The initial clinical diagnosis was considered to be acute FM, but not acute myocardial infarction. Therefore, we did not urgently perform coronary angiography with the support of ECMO.

The patient’s vital signs in the later stage of hospitalization were relatively stable, but his cardiac function remained diminished. And then, it was very important to went out of the ICU to further improved the inspections (e.g., coronary ultrasound, coronary CTA, and cardiac MRI). The congenital abnormal origin of the left coronary artery was detected using transthoracic echocardiography, and confirmed with cardiac CTA. Enhanced cardiac MRI showed the acute subendocardial myocardial infarction of the left ventricle, confirming the etiological diagnosis.

The anomalous origin of the left coronary artery arising from the right coronary sinus is rare, approximately 0.01–0.04% (2). The abnormal coronary artery originating from the contralateral coronary sinus and running between the aorta and pulmonary artery with intramural deviation, is related to sudden death (grade III) (11). The patient had a history of syncope during exercise and accompanied by a significant increase in troponin I (up to 190 ng/ml). The pathogenesis of the patient may be that the compensatory cardiac systolic function was enhanced during vigorous activity, and the gap between the dilated aorta and the pulmonary artery became smaller, resulting in a exacerbated clamping effect, squeezing the LM, also known as “coronary artery sandwich anomaly” (12). This can lead to complete occlusion of the LM, causing acute subendocardial myocardial infarction and fatal malignant arrhythmias.

The presence of a AOCA can be suspected in the case of a young individual with ischemia-like symptoms (13). Coronary CTA is currently considered the gold standard, and cardiac MRI is becoming an alternative. Because of coronary angiography’s invasiveness, relatively low spatial resolution, and lack of 3-dimensional images, it has been progressively replaced by coronary CTA (13). Transthoracic echocardiography is considered a key examination in the diagnostic workup of AOCAs in children, in whom optimal acoustic windows commonly allow the visualization of coronary ostia without radiation exposure (13). Although coronary angiography is not the gold standard for diagnosing AOCA, lack of early coronary angiography in this case is still a limitation of this case. Coronary angiography remains critical in the diagnosis and differential diagnosis of patients with cardiogenic shock. Echocardiography in children with cardiogenic shock, attention should be paid to the origin of coronary arteries. ECMO and IABP can support the circulation of patients, so that patients can survive the period of malignant arrhythmia and severe shock, and the definitive diagnosis can be identified and treated later. This suggests that it is important to investigate the causes of the critically ill patients’ condition, but saving lives remains the first priority. For adolescent patients, sudden cardiorespiratory arrest, especially when related to increased activity, should be carefully ruled out to exclude congenital coronary artery origin and abnormal course, to reduce the occurrence of subsequent malignant events.

## Conclusion

The successful treatment of this patient was attributed to the rapid ECMO assisted circulation, supplemented by IABP. Establishing clear diagnosis is a process of multidisciplinary joint diagnosis, which provides a reference for clinicians when encountering similar cases.

## Data Availability Statement

The original contributions presented in the study are included in the article/supplementary material, further inquiries can be directed to the corresponding authors.

## Ethics Statement

Written informed consent was obtained from the individual(s) and minor(s)’ legal guardian/next of kin, for the publication of any potentially identifiable images or data included in this article.

## Author Contributions

XX, PX, and RZ drafted the manuscript and contributed to the case collection. XW, YC, and XH provided figures and formalized the manuscript. HL and JY reviewed the drafts and approved the final manuscript as submitted. All authors approved the submitted version.

## Conflict of Interest

The authors declare that the research was conducted in the absence of any commercial or financial relationships that could be construed as a potential conflict of interest.

## Publisher’s Note

All claims expressed in this article are solely those of the authors and do not necessarily represent those of their affiliated organizations, or those of the publisher, the editors and the reviewers. Any product that may be evaluated in this article, or claim that may be made by its manufacturer, is not guaranteed or endorsed by the publisher.
